# Implication of the p53-Related miR-34c, -125b, and -203 in the Osteoblastic Differentiation and the Malignant Transformation of Bone Sarcomas

**DOI:** 10.3390/cells9040810

**Published:** 2020-03-27

**Authors:** Camille Jacques, Robel Tesfaye, Melanie Lavaud, Steven Georges, Marc Baud’huin, François Lamoureux, Benjamin Ory

**Affiliations:** INSERM, Bone sarcomas and remodeling of calcified tissues, Nantes Université, UMR 1238, F-44000 Nantes, France; cjacques44000@gmail.com (C.J.); robel.tesfaye@etu.univ-nantes.fr (R.T.); melanie.lavaud@etu.univ-nantes.fr (M.L.); Steven.Georges1@univ-nantes.fr (S.G.); marc.baudhuin@univ-nantes.fr (M.B.); francois.lamoureux@univ-nantes.fr (F.L.)

**Keywords:** bone, osteosarcoma, microRNAs

## Abstract

The formation of the skeleton occurs throughout the lives of vertebrates and is achieved through the balanced activities of two kinds of specialized bone cells: the bone-forming osteoblasts and the bone-resorbing osteoclasts. Impairment in the remodeling processes dramatically hampers the proper healing of fractures and can also result in malignant bone diseases such as osteosarcoma. MicroRNAs (miRNAs) are a class of small non-coding single-strand RNAs implicated in the control of various cellular activities such as proliferation, differentiation, and apoptosis. Their post-transcriptional regulatory role confers on them inhibitory functions toward specific target mRNAs. As miRNAs are involved in the differentiation program of precursor cells, it is now well established that this class of molecules also influences bone formation by affecting osteoblastic differentiation and the fate of osteoblasts. In response to various cell signals, the tumor-suppressor protein p53 activates a huge range of genes, whose miRNAs promote genomic-integrity maintenance, cell-cycle arrest, cell senescence, and apoptosis. Here, we review the role of three p53-related miRNAs, miR-34c, -125b, and -203, in the bone-remodeling context and, in particular, in osteoblastic differentiation. The second aim of this study is to deal with the potential implication of these miRNAs in osteosarcoma development and progression.

## 1. Introduction

Bone is a highly dynamic structure that has both biomechanical and biologic functions, as it protects the inner organs, furnishes muscle attachment, provides calcium and phosphorus reservoirs, and assumes hematopoietic functions. Bone remodeling is an important process, starting in the embryo and continuing throughout the entire life of vertebrates. It allows the bone-mass to stay constant through the antagonist-balancing activities of the two kinds of bone-related cells: the bone-forming osteoblasts and the bone-resorbing osteoclasts. Impairments in these balanced activities may result in osteo-condensing bone pathologies such as osteopetrosis or osteolytic metabolic lesions, like osteoporosis, and can also be the origins of bone cancers. Malignant bone diseases are rare pathologies fueled by the establishment of a vicious cycle between the tumor cells and the above-mentioned bone-specialized cells. With an incidence of four to five cases per million and a peak at around 18 years of age, osteosarcoma is by far the most common type of these neoplasms [1,2]. The suspected initiated cell in this pathology is the osteoblast [3], and even if, to date, no specific genetic lesion has been uncovered to characterize this pathology, the locus of the tumor suppressor gene *p53* is altered in about 50% of patients [4,5]. This cancer is most often localized on the metaphysis of the long bones of the extremities, namely, the distal femur, the proximal tibia, and the proximal humerus [6]. Despite recent progress in the therapeutic management of osteosarcomas, the survival rates have not increased in two decades. Thus, to improve the outcome of this pathology, a better understanding of the mechanisms governing the osteoblastic differentiation, the bone-remodeling processes, and, more generally, the carcinogenesis of this cancer are still needed. Worthy of note is that it is now well-established that epigenetic mechanisms such as those implicating the small regulatory microRNAs (miRNAs) are of paramount importance to the control of such processes and to the consequent initiation and malignant progression of osteosarcomas.

Since the discovery of the first miRNA, *lin-4,* implicated in the development of the microscopic worm *C. elegans* [7], it has been well-established that these evolutionarily conserved molecules add a novel complex epigenetic regulation layer to the control of gene expression. MiRNAs are small non-coding RNAs of about 22–24 nucleotides in length that disrupt gene expression of messenger RNAs (mRNAs) through the base-pairing in their 3′-untranslated regions (UTR). Depending on their target sequence homology, they induce either translational repression or mRNA degradation and, consequently, lower the levels of target proteins. Bioinformatics analysis reveals that more than 30% of human genes could be regulated by miRNAs [8]. Because a unique miRNA is sometimes able to target more than a hundred of different mRNAs [9], such regulators can powerfully balance complex networks and constitute critical control nodes in response to the cell environment. In recent years, intensive research has highlighted their implication in various biological processes such as proliferation, cell cycle control, differentiation, or apoptosis. Additionally, they were found to be aberrantly deregulated in a number of diseases, including cancers. Evidence of a relevant implication of miRNAs in cancers was reported for the first time in 2002, after the observation that the miR-15a and -16-1 were often down-regulated or deleted in chronic lymphocytic leukemia cancers [10]. It is worth noting that some miRNAs down-regulate genes with oncogene properties and have, in this case, a tumor suppressor role. On the other hand, some others directly target tumor-suppressor genes and are called oncomiRs. To effectively mediate their inhibitory role, several maturation steps of these molecules are needed. The RNA polymerase II (RNA pol II) is the first player in miRNA biogenesis, allowing for the transcription of a hairpin-structured primary-transcript (pri-miRNA). The latter is then cleaved by the endonuclease III complex DROSHA/DGCR8, leading to a 70-nucleotide length pre-miRNA. The generated pre-miRNA is then exported out of the nucleus by the Exportin-5 before undergoing a second maturation step assumed by the endoribonuclease DICER, producing the mature miRNA. The latter is finally carried by the AGONAUTE slicer-complex to form an active inhibitor-featured structure termed the miRNA-induced silencing complex (RISC). Considering the fact that the miRNA’s promoters bear a close resemblance to those of the protein-coding genes, the expression of these small regulators is modulated by the same regulating processes and, thus, is under the control of a plethora of transcription factors such as p53.

The *TP53* gene, encoding the p53 protein, is certainly the most famous tumor-suppressor gene in the field of cancer biology due mainly to its genome-safeguard properties. The p53 family is composed of three sequence-specific transcription factors, p53 itself, p63, and p73, regulating the expression of a variety of direct target genes implicated in DNA repair, the induction of cell-cycle arrest, cell senescence, and apoptosis [11,12]. The tumor-suppressor functions of p53 are, moreover, supported by the fact that over 50% of human cancers display mutation or inactivation in this gene [13,14]. In addition, such mutations or deletions have been associated with Li-Fraumeni syndrome and a predisposition to developing a wide range of tumors including osteosarcomas [15,16]. Various cellular stresses such as DNA damage, hypoxia, nutrient deprivation, or γ-irradiation serve as stimuli that are integrated by p53, allowing for its stabilization and, consequently, ensuring both its nuclear translocation and its binding to the promoters of its target genes [17].

**Figure 1:**
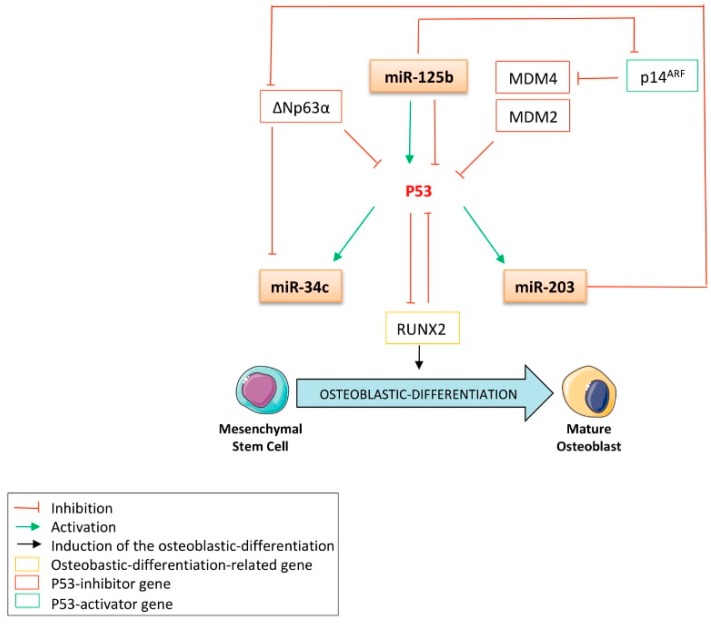
The relationship among p53, the p53 activators, the p53 inhibitors, and the miR-34c, -125b and -203 is essential to control the osteoblastic differentiation. MDM2: mouse double minute 2 homologue; MDM4: mouse double minute 4 homologue; RUNX2: runt-related transcription factor 2.

In the bone-related concerns of this review, the role of p53 in controlling both apoptosis and cell growth has been shown to be crucial to the preservation of bone homeostasis. It was reported that p53-mutated mice, in which p53 activity is increased, displayed a particular early-aged osteoporotic phenotype [18]. On the other hand, the p53^-/-^ mice model was reported to harbor a high bone-mass phenotype. These data are in accordance with other publications reporting the anti-osteoblastic-differentiation role of p53 [19]. It is now well established that p53 counteracts bone remodeling, primarily by impairing the osteoblastic differentiation program driven by two pivotal osteoblastic-related transcription factors: RUNX2 (runt-related transcription factor 2) and Osterix [20]. Both are essential cooperating factors in osteoblastic differentiation, and the expression of the zinc fingers that contain factor Osterix is controlled by RUNX2, as its expression was abrogated in RUNX2-null mice [21]. The role of p53 in osteoblastic differentiation is due not only to its transcription factor properties but it also has been demonstrated to repress the expression of Osterix in a promoter-binding independent manner [20]. In the same study, investigators showed that p53^-/-^ mice display increased bone mineral density and that p53^-/-^ osteoblasts exhibit accelerated differentiation. By using MDM2 (mouse double minute 2 homologue)-conditional Col3.6-Cre mice, in which the conditional deletion of the p53 inhibitor MDM2 is allowed in osteoblasts, Lengner et al. showed that the negative regulation exerted by MDM2 on p53 is required for proper osteoblastic differentiation [22]. Additionally, in these cells, alkaline-phosphatase (ALP), osteocalcin (OCN), and type I collagen (Coll-I) expression did not display the increase normally observed in wild-type osteoblast progenitor cells during maturation. Another study reported that embryonic fibroblasts from p53^-/-^ mice express high levels of RUNX2 and Osterix as well as exhibit higher basal- and BMP4-induced ALP activity as compared to normal fibroblasts [23]. In addition, the siRNA-mediated or genetic null mutation-mediated knock-down of p53 in osteoblasts from mice calvarias results in an increased expression of RUNX2 [24]. On the other hand, the induction of p53 by either γ-irradiation, by inhibiting the expression of MDM2 by treatment with its pharmacological inhibitor Nutlin-3, or through the conditional expression of its cellular inhibitor p14/p19^ARF^ all result in a decrease in the expression of RUNX2 at the protein level. Interestingly, a feed-back regulation loop was also demonstrated by Ozaki et al., who found that RUNX2 was a p53 inhibitor (Figure 1) [25].

Beyond the direct transcriptional activity of p53, its ability to induce the expression of miRNAs confers on it a novel, indirect, negative regulation power over gene expression. It thus broadens our knowledge about its tumor-suppressive activity, especially as it has been well documented that several p53-related miRNAs are implicated in cancer outset, promotion, and extent. In addition, the recent breakthroughs in the field of cell-fate-determination research have progressively integrated the miRNAs as key modulators of osteoblastic differentiation (e.g., miR-138 and -206, which have already been described as inhibitors of this process [26,27]). In line with this consideration, this review aims to focus on three interesting p53-related miRNAs: miR-34c, -125b, and -203. Their direct and indirect involvement in both osteoblast biology and tumorigenesis are studied and provide a novel glimpse at the indirect implications of p53 in the development of osteosarcoma.

## 2. The Expression of miR-34c and -203 Is Controlled by p53

### 2.1. MiR-34c

#### 2.1.1. MiR-34c and Cancer: Generalities

MiR-34c belongs to the miR-34 family, which encompasses three members of vertebrates: miR-34a, transcribed from a large transcriptional unit on chromosome 1p36, and miR-34b and -34c, which are both co-transcribed from the chromosome 11q23 [28]. An expansive body of literature shows that miR-34c is often subjected to severe down-regulation in various cancer models, such as nasopharyngeal carcinomas [29], laryngeal carcinomas [30], colon adenocarcinomas [31], uveal melanomas [32], gliomas [33], prostate cancers [34], and osteosarcomas [24]. Such down-regulation could be partially explained by epigenetic mechanisms, as the CpG islands of miR-34c’s promoters were found to be hypermethylated in, for instance, colorectal cancers [35,36], gastric cancers [37], and myeloproliferative neoplasms [38]. In addition, the aberrant hypermethylation of the miR-34c’s promoter is correlated with a high probability of the recurrence of stage-1 non-small-cell lung cancer (NSCLC) and is also associated with poor overall survival and low disease-free survival [39]. Interestingly, some other studies report that, on the other hand, miR-34c is overexpressed in squamous cell carcinomas of the tongue [40], high-grade cervical intraepithelial neoplasms [41], and cancer stem cells of hepatocellular carcinomas [42]. Nevertheless, as quiescence is a bona fide stem cell property, the overexpression of this miRNA in the latter models could finally contribute to sustaining the dormant steady state of those cells and, thus, impair the pro-proliferative pathways instead of serving as an oncogene signature. Nonetheless, all these contradictory studies remind us that, given the multiple targets of a single miRNA, its role in whole integrated systems depends on the cell type, the cellular context, and, obviously, environmental factors. In this regard, as miR-34c seems to be a key player in the maintenance of the normal cell’s phenotype, its expression must be subjected to tight control.

#### 2.1.2. The Expression of miR-34c Is Directly Induced by p53

A large body of evidence reports that the expression of miR-34c is fine-tuned by several tumor-suppressive pathways, such as the p53 pathway (Figure 1). By performing chromatin immunoprecipitation (ChIP) assays after the induction of DNA damage in wt- or p53^-/-^ murine embryonic fibroblasts, He et al. became the first to report that miR-34′s family is a direct transcriptional target of p53 [43]. This group found that the promoter of miR-34′s family contains a palindromic sequence matching the canonical p53-binding site. Such results were soon afterward extended in colon adenocarcinomas cells by Bommer et al., who characterized the direct role of p53 in the transcription of miR-34c by monitoring miR-34b/c promoter-activity via luciferase reporter assays in cells transfected with wt or mutant p53 [44]. They found that the luciferase activity was significantly enhanced only after transfection of the wt form of p53. In addition, Corney et al. performed miRNA microarrays and RT-qPCR analysis and reported a twelve-fold decrease in miR-34c expression after the conditional inactivation of p53 in cells from the mouse ovarian epithelium [45]. These investigators also found that the expression of this miRNA is lost in human ovarian-adenocarcinoma p53-null cells. Such results are in accordance with those of Kumamoto et al., who found that treating fibroblasts with the MDM2 inhibitor Nutlin-3a increases the expression of p53 and the expression of its target genes, including miR-34c, thus inducing cell senescence [46]. Additionally, the authors showed that the up-regulation of this miRNA does not occur in cancer cell lines harboring neither a null nor a mutant p53 protein. He et al. further confirmed these results in a bone cancer model, as both γ-irradiation or an Adriamycin treatment strongly induce miR-34c expression in the U2OS p53-wt osteosarcoma cell line but not in the SaOS-2 p53-null one [47]. The authors also showed that transfecting this miRNA in these cell lines induced a G1 phase cell-cycle arrest, while annexin-V staining revealed a more potent induction of apoptosis in wt-p53 cells than in p53-null cells. More recently, the ChIP assays from Fabbri et al. confirmed the p53-transactivating activity on miR-34c in a leukemia model [48]. Another interesting proof of the miR-34c’s bona fide role as a tumor suppressor is that it is inhibited by the p53 inhibitor ∆Np63α. Indeed, the study of Antonini et al., performed in a primary mouse keratinocyte model, revealed that ∆Np63α directly binds to the p53-consensus binding site of the miR-34c promoter, leading to the inhibition of the activity of this promoter [49]. Together, this data supports the notion that miR-34c’s family is a p53 transcriptional target and advocates for the tumor-suppressive role of this miRNA, which will be further developed in this review.

### 2.2. MiR-203

#### 2.2.1. MiR-203 and Cancer: Generalities

Similar to miR-34c, miR-203 is often subjected to deregulation in several malignancies and is undoubtedly linked to crucial processes affecting cancer induction and spreading. MiR-203 is reported to be down-regulated in esophageal cancers [50,51], melanomas [52], basal cell carcinomas [53], cervical intraepithelial neoplasias [41], and bone metastasis from prostate cancers [54]. In addition, the promoter region of this miRNA was found to be hypermethylated in multiple myelomas [55], rhabdomyosarcomas [56], hepatocellular carcinomas [57], oral squamous cell carcinomas [58], prostate cancers [59], myeloproliferative neoplasms [38], cervical cancers [60], nickel-transformed bronchial epithelial cells [61], and hematopoietic malignancies [62]. By contrast, the expression of miR-203 is increased in colon adenocarcinomas [31] and bladder cancer models [63]. Of note, its role has been well-studied throughout the course of epidermal differentiation, as it is one of the most overexpressed miRNAs in keratinocytes [64].

#### 2.2.2. P53 and miR-203, an Indirect Interplay: The Expression of miR-203 Is Indirectly Induced by p53 and miR-203 also Indirectly Regulates p53

Although a plethora of evidence indicates that p53 is an inducer of the miR-34c family’s expression, only an indirect regulation of miR-203 by p53 has been reported. Suzuki et al. were the first to highlight this indirect regulation, as they found that a Doxorubicin treatment increases p53 expression as well as that of miR-203 in the human colon cancer cell line HCT116 (Figure 1) [65]. In accordance with these results, a down-regulation of miR-203′s expression was observed after either a p53 knock-down or a p53 degradation by the human papillomavirus type 16 (HVP16) oncoprotein E6, in a human foreskin fibroblast model [66]. This group has also shown that the introduction of the wt-p53 gene into the p53-null human lung cancer cell line H1299 leads to an increase in miR-203, whereas a p53-mutated introduction does not. These results thus confirm that the expression of miR-203 is dependent on the proper transcriptional activity of p53. In addition, the expression of miR-203 correlates with the level of DNA-damage response induced by either Ultra violet radiation [67] or doxorubicin treatment [66], both of which are known to induce the activity of p53. More recently, this regulation was supported by the fact that cisplatin promotes cell death through the induction of miR-203′s expression in a p53-dependent manner [68]. On the other hand, it is noteworthy that primary human lung fibroblasts transfected with the p53-inactivating peptide GSE56 display an increased expression of miR-203, suggesting, in this model, the repressive role of p53 on the expression of this miRNA [69]. Interestingly, Yi et al. highlighted another indirect feedback mechanism by which miR-203 controls the expression of p53 itself [64]. This group demonstrated that the direct targeting capabilities of miR-203 toward the p53 inhibitor p63 are conserved across the vertebrates, as reduced luciferase activity was obtained in a reporter construct bearing the p63-3′UTR of human, mouse, and zebrafish (Figure 1). The interesting, indirect interplay between miR-203 and both p53 and the miRNA’s target genes, which will be developed further in this review, give this miRNA a central place in the context of bone remodeling.

### 2.3. MiR-125b

#### 2.3.1. MiR-125b and Cancer: Generalities

Located on the chromosome 11q24.1, miR-125b has been well-studied in the past. Nevertheless, its role in cancer is not well understood, as several contradictory studies report either oncogene or tumor-suppressor properties, depending on the models. It is underexpressed in medulloblastomas [70], cutaneous squamous cell carcinomas [71], osteosarcomas [72], melanomas [73], ovarian cancers [74], and the hyperproliferative keratinocytes found in psoriasis lesions [75]. On the other hand, it was also reported that reconstituted mice with modified bone marrow in which the expression of miR-125b was enforced exhibited clear myeloproliferative disorders, increasingly progressing to myeloid leukemia [76]. Furthermore, Iida et al. sustained its oncogenic properties, as they found that this miRNA was up-regulated in both doxorubicin-resistant Ewing sarcoma cell lines and human tumor samples that escaped from chemotherapy [77]. Down-regulating this miRNA in such resistant cell lines sensitizes them to doxorubicin, vincristine, and etoposide, thus suggesting its implication in the regulation of a multidrug-resistance pathway. In addition, its increased expression throughout the course of human mesenchymal stem cell (MSC) differentiation into the osteoblastic lineage also implicates miRNA-125b in this process [78].

#### 2.3.2. MiR-125b Represses the Expression of p53

To ensure a robust and proper response to the cellular environment and to suppress tumors, both the expression and the activity of p53 must be tightly controlled. Thus, several transcriptional repressors, such as the p53-related member **∆Np63α**, were previously reported to have a dominant-negative role on p53 (Figure 1) [79]. In addition, the **MDM2** and **MDM4** ligases are two well-known p53-post-translational inhibitors, as they induce its ubiquitination-mediated degradation [80]. Moreover, over the last decade, it has become obvious that the expression of p53 is also subjected to epigenetic mechanisms, in particular, those coordinated by the miRNAs. Additionally, miR-125b is worthy of attention in the context of this review not only because of the extensive evidence of its direct inhibitory role on p53′s expression but also because of its intriguing repressive functions on osteoblastic differentiation. Le et al. were the first to report that this miRNA directly targets p53′s 3′UTR in human lung fibroblasts and in the zebrafish brain [81]. In addition, this group demonstrated that the expression of miR-125b decreases after either camptothecin- or γ-irradiation-induced DNA damage, in accordance with the rapid p53 increase observed in response to such injuries. It was further confirmed by Iida et al. that p53 was a direct target of miR-125b in Ewing sarcoma cells [77]. More recently, Amir et al. reported another indirect mechanism by which miR-125b controls the expression of p53 in a prostate cancer model [82]. This group reports that this miRNA directly targets the MDM2 inhibitor **p14^ARF^** and consequently inhibits the expression of p53 and its target genes, p21 and PUMA (Figure 1). Functionally, it was found that miR-125b represses the p53-induced apoptosis, as its overexpression in the human neuroblastoma SH-SY5Y cell line consequently reduces the expression of p21 and Bax, another p53-target gene [81].

#### 2.3.3. MiR-125b Induces the Expression of p53

Even if miR-125b appears as a p53 inhibitor, its relationship with the guardian of the genome is not clear-cut. It is important to take note of another study reporting that a stable increase of miR-125b expression in cutaneous malignant melanoma cells leads to an increase in the expression of p53 at the protein level, further inducing cell senescence (Figure 1) [83]. The dual role of miR-125b on the expression of p53 is obviously highly dependent on the expression level and/or activity of p53 inhibitors and highlights, once again, the complexity and necessity of fine-tuning the expression of p53.

## 3. p53-related miRNAs Inhibit Osteoblastic Differentiation through Their Ability to Impair the Expression of Several Bone-Related Factors

As miRNAs are implicated in several biological processes, it seems obvious that they are also implicated in bone remodeling and the consequent pathologies associated with defects in this mechanism [84,85]. A better understanding of the implication of miRNAs in osteoblastic differentiation is essential to give rise to future clinical applications and hopefully improve the outcome of bone fractures and bone malignancies such as osteosarcoma.

### 3.1. The Osteoblastic Differentiation of Multipotent Mesenchymal Stem Cells Is Controlled by Several Crucial Factors and Pathways

**Figure 2:**
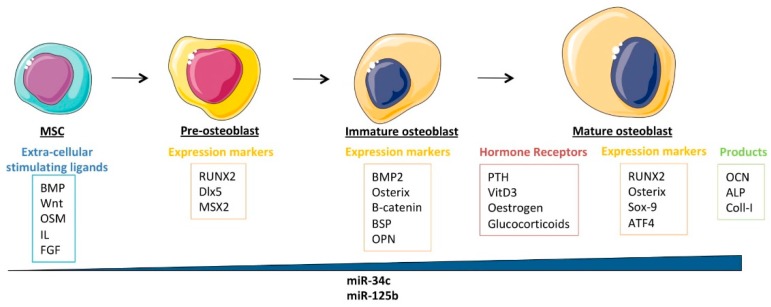
The osteoblastic-differentiation process; **ALP**: alkaline phosphatase; **ATF4**: activating transcription factor 4; **BMP**: bone morphogenetic protein; **BSP**: bone sialoprotein; **Coll-I**: type I collagen; **FGF**: fibroblast growth factor; **IL**: interleukin; **MSC**: mesenchymal stem cells; **MSX2**: Msh homeobox 2; **OCN**: osteocalcin; **OPN**: osteopontin; **OSM**: oncostatin M; **PTH**: parathormone; **RUNX2**: runt-related transcription factor 2. The blue triangle represents the increased miRNA-expression level found in the cells during the course of the differentiation.

Multipotent mesenchymal stem cells (MSCs) are subjected to osteoblast differentiation through several stimulating extracellular ligands such as bone morphogenetic proteins (BMPs), Wnts, oncostatin-M (OSM), interleukins (IL), and fibroblast growth factors (FGFs) (Figure 2). They first differentiate into pre-osteoblasts, which are characterized by the expression of Runt-related transcription factor 2 (RUNX2), distal-less homeobox 5 (Dlx5), and Msh homeobox homologue 2 (MSX2). They then differentiate into immature osteoblasts, expressing bone morphogenetic protein 2 (BMP2), Osterix, β-catenin, bone sialoprotein (BSP), and osteopontin (OPN). Mature osteoblasts are osteocalcin, alkaline phosphatase, and type-I collagen producers, allowing them to form mineralized bones [86]. These cells also express several osteoblast differentiation-related hormone receptors, such as the receptors for parathormone (PTH), 1α,25-dihydroxyvitamin D_3_, estrogen, and glucocorticoids. In addition to RUNX2 and Osterix, the mature osteoblasts express several key transcription factors such as SOX9 and activating transcription factor 4 (ATF4), all required for their differentiation into the bone-associated cell lineage. It was shown that all these factors physically associate to cooperate with RUNX2 and Osterix in their transcriptional capabilities [87], suggesting that impairment in these interactions could reduce the potential of cellular differentiation. In return, these transcription factors are controlled by a series of tightly regulated pathways, which are also often deregulated in cancers, including osteosarcomas. These pathways include, for instance, the Notch, the Sonic/Hedgehog, the Wnt, the TGF-β, and the BMP and will be detailed further in this review, as impairment of them might have an indirect negative effect on osteoblastic differentiation (Figure 2).

#### 3.1.1. The p53-Related miRNAs Regulate the Early Stages of Osteoblastic Differentiation by Controlling the Pre-Osteoblast-Specific Factor RUNX2

##### RUNX2

**Figure 3:**
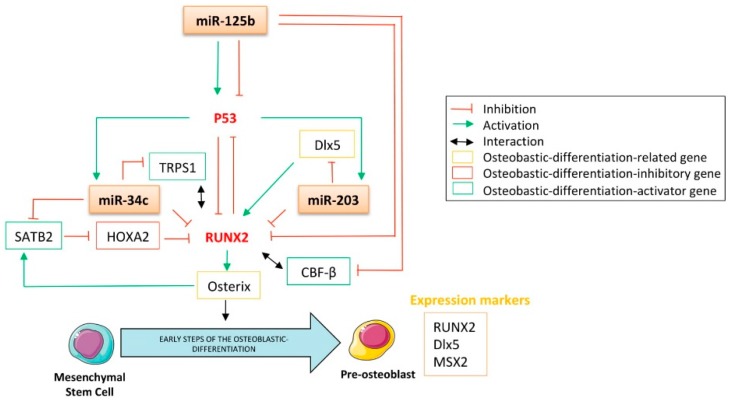
The direct targeting of RUNX2 and Dlx5 by the p53-related miRNAs involved them in the control of the early steps of the osteoblastic differentiation. CBF-β: core-binding factor β; MSX2: Msh homeobox 2; RUNX2: runt-related transcripion factor 2; SATB2: Special AT-rich sequence binding protein 2; TRPS1: Tricho-rhino-phalangeal syndrome 1.

MiRNAs are crucial regulators of bone formation through their ability to attenuate signaling pathways and transcription factors’ expression implicated either in osteoblastic differentiation or in their function. Among all the transcription factors related to this process, RUNX2 is one of the most-studied. It belongs to the runt-related sequence-specific transcription factor family, which encompasses three members: RUNX1, RUNX2, and RUNX3. Their runt DNA binding domain is homologous with the drosophila pair-rule gene *runt* [88] and confers on them the ability to associate with the co-activator core-binding factor β (CBF-β) [89]. Even though RUNX2 is involved in the tightened regulation of various cellular processes, including development, tumorigenesis, and differentiation, its role has been particularly studied in osteoblastic and chondrocytic differentiation, especially in the early stages of these processes. Beyond the differentiation process, evidence also reports its implication in the proper function of osteoblasts, as it is required for both bone formation and mineralization [90]. For instance, it was reported that RUNX2^-/-^ mice display a lack of osteoblasts [91]. Additionally, RUNX2 was found to be overexpressed in chemoresistant osteosarcomas [92], non-small cell lung cancers (NSCLCs) [93], and breast cancers [94], thereby raising the legitimate question of its role as a tumor-driving transcription factor. The latter has not yet been clearly demonstrated but it was reported that RUNX2 contributes to the development of T-cell lymphoma through its cooperation with the oncogene c-Myc [95]. There is no evidence in the literature that p53 directly modulates the expression of RUNX2, especially as no p53-canonical binding site was found in the RUNX2 promoter sequence. Nonetheless, regarding p53′s inhibitory effect on osteoblastic differentiation, the p53-regulated miRNAs able to target RUNX2 might largely be implicated in such features. Indeed, miR-34c was reported to repress RUNX2 by direct interaction, as assessed by reporter assays after modulation of the miRNA’s expression level by mimic- or anti-miRNA transient transfections in mouse MC3T3-E1 osteoblasts (Figure 3) [96]. Increasing the expression of miR-34c in these cells significantly reduces the RUNX2 protein expression level but also impacts the osteoblastic differentiation markers OCN and OPN. In addition, such an increase has a functional effect on reducing ALP activity. The direct interaction between miR-34c and RUNX2-mRNA was further supported by Van der Deen’s work on an osteosarcoma model [24], in which the weak expression level of this miRNA in SaOS-2 and U_2_OS osteosarcoma cell lines was also reported. Interestingly, Wei et al. found that the expression level of miR-34c increases during the course of the differentiation of osteoblasts from mouse calvaria [97]. By generating a transgenic mice model overexpressing miR-34c in the osteoblasts and displaying a lower mineralized bone volume over the total volume (BV/TV) compared to normal mice, this group highlighted the negative impact of miR-34c on osteogenesis. Interestingly, this group also showed that bone OCN and BSP levels were affected by the enforced expression of this miRNA in osteoblasts. Surprisingly, such conditions did not alter the expression levels of RUNX2, ATF4, Osterix, and the receptor activator of nuclear factor κb ligand (RANKL), an osteoblast- and osteoclast-activating cytokine.

Both miR-34c and miR-203 were reported to repress RUNX2′s expression through direct interaction with the 3′UTR of the RUNX2 messenger (Figure 3) [54]. Functionally, overexpression of this miRNA in the PC3 prostate carcinoma cell line significantly reduces the expression of RUNX2 at the protein level. Interestingly, it was reported that through its RUNX2 targeting capabilities, miR-203 was able to impair the progression of breast cancer as well as reduce its bone-associated metastases [98].

Interestingly, contradictory data was published concerning the role of miR-125b in RUNX2-expression modulation. For instance, Chen’s work showed that the expression of RUNX2 increased in the thoracic aorta of rats suffering from chronic kidney disease and corresponded to a reduced expression of vascular miR-125b in this model [99]. The chronic-kidney-disease animals also displayed increased calcification of the aortic arch, negatively correlated with the miR-125b level in the thoracic aorta. These data sustain the inhibitory role of miR-125b on vascular calcification, possibly through its ability to modulate the expression of RUNX2, even if no direct targeting was exhibited here. On the other hand, another group showed no modulation in RUNX2, OPN, and ALP gene expressions subsequent to the gain or loss of miR-125b in MSCs [78]. Interestingly, these investigators reported an increase in the expression level of this miRNA during the osteoblastic differentiation of such cells. In addition, it is now well established that CBF-β physically associates with RUNX2 to increase its DNA affinity [100], and miR-125b was precisely reported as inhibiting CBF-β-mediated osteogenesis through direct CBF-β-targeting (Figure 3) [101]. In addition, through the regulation of the proliferation of mouse mesenchymal stem cells, an inhibitory effect of miR-125b on BMP-4-induced osteoblastic differentiation was highlighted [102]. This study reported a decrease in the expression of the OCN transcript, as well as reduced ALP activity after miR-125b’s transfection into ST2 and NRG stem cells. By assessing the matrix mineralization capabilities of calcifying human coronary artery smooth muscle cells (HCASMCs), their RUNX2 mRNA expression level, as well as their ALP mRNA expression level and activity, Goettsh et al. found that the inhibition of endogenous miR-125b promotes osteogenic differentiation [103]. In addition, investigators noticed a significant up-regulation of Osterix at both mRNA and protein levels when miR-125b was inhibited in calcifying HCASMCs.

Additionally, even if RUNX2 is a key player in osteoblastic differentiation, it does not drive the entire process by itself [104]. Thus, in a differentiation control context, the multi-targeting capabilities of the above-mentioned p53-related miRNAs seem to be of particular interest. Numerous other nuclear factors also cooperate with RUNX2 to elicit osteoblastic differentiation, for example, special AT-rich sequence binding protein 2 (SATB2), the homeobox transcription factor Dlx5, and the tricho-rhino-phalangeal syndrome I factor TRPS1 [105]. Their targeting by these miRNAs and the consequent effect on both the expression and activity of RUNX2 might impede osteoblastic differentiation (Figure 3).

##### Special AT-Rich Sequence Binding Protein 2 (SATB2)

**Special AT-rich sequence binding protein 2** (**SATB2**) is a member of the special AT-rich binding protein family, characterized by its ability to bind to the so-called nuclear matrix attachment regions (MARs) to further modulate the chromatin structure and, consequently, impact gene transcription. It has been reported to be an osteoblastic differentiation marker [106,107], and Osterix was shown to directly regulate its transcription [108]. The effect of SATB2 on the regulation of osteoblastic differentiation is partially explained by its inhibitory effect on the RUNX2 inhibitor Hoxa2, consequently resulting in increased activity of both RUNX2 and activating transcription factor 4 (ATF4) [109,110]. Wei et al. reported that miR-34c directly binds to the 3′UTR of SATB2 to down-regulate its expression, resulting in an inhibition of both the proliferation and the differentiation of mice osteoblasts (Figure 3) [97].

##### The Homeodomain-Containing Transcription Factor Dlx5

The homeodomain-containing transcription factor Dlx5 is a member of the evolutionary conserved Dlx homeobox gene family, which encompasses six genes organized into bi-genes clusters: Dlx1/2, Dlx5/6, and Dlx3/7. Such genes are homologous to the *Distal-less* (*Dll*) gene of drosophila and are required during organogenesis and bone differentiation. It was shown that Dlx5 is expressed in all the skeletal structures of mid-gestation mice embryos after first cartilage formation [111]. Additionally, it was reported that mice lacking Dlx5 expression display craniofacial defects, delayed ossification of the roof of the skull, and abnormal osteogenesis [112]. It is noteworthy that human miR-203, down-regulated in bone metastatic prostate cancer cells, binds to the 3′UTR of Dlx5 [54]. As Dlx5 is a well-known inducer of RUNX2 [113,114] and, therefore, an important regulator of osteogenesis [115], miR-203 represses RUNX2′s expression by an additional indirect targeting, resulting in the blockade of osteoblastic differentiation (Figure 3). Interestingly, another study reported that bone-metastasis cells from prostate cancer also express RUNX2 [116]. Furthermore, it was here demonstrated that through its ability to target both RUNX2 and Dlx5, miR-203 decreases the expression of osteopontin and osteocalcin in prostate cancer cells. Such a decrease affects the osteo-mimetic features of these cells, normally allowing them to grow and develop in bones, and here suggesting the implication of this miRNA in the metastatic process [54].

##### Tricho-Rhino-Phalangeal Syndrome I (TRPS1)

The chondrogenic GATA-like transcription factor tricho-rhino-phalangeal syndrome I (TRPS1) is another key effector of skeletal development. Mutations of this gene are responsible for tricho-rhino-phalangeal syndrome, which notably includes skeletal abnormalities [117]. This gene is overexpressed in breast [118] and colon cancers [119]. This gene is also used as a biomarker in this model, as its expression level correlates with both the lymph node metastasis invasion and the pathological stage. Surprisingly, it was reported that this factor has the ability to directly bind to the OCN promoter to act as a repressor of its transcription [120]. A recent work reported that miR-34c targets **TRPS1**, resulting in the blockade of both osteoblastic and chondrogenic differentiation and further supporting the tumor-suppressing role of miR-34c (Figure 3) [96].

## 4. p53-Related miRNAs Regulate Osteoblastic Differentiation by Targeting Pleiotropic Pathways

### 4.1. The p53-Related miRNAs’ Interplay with the Notch, Wnt/β-catenin, and Sonic/Hedgehog (HH) Pathways

#### 4.1.1. The Notch Pathway

**Figure 4:**
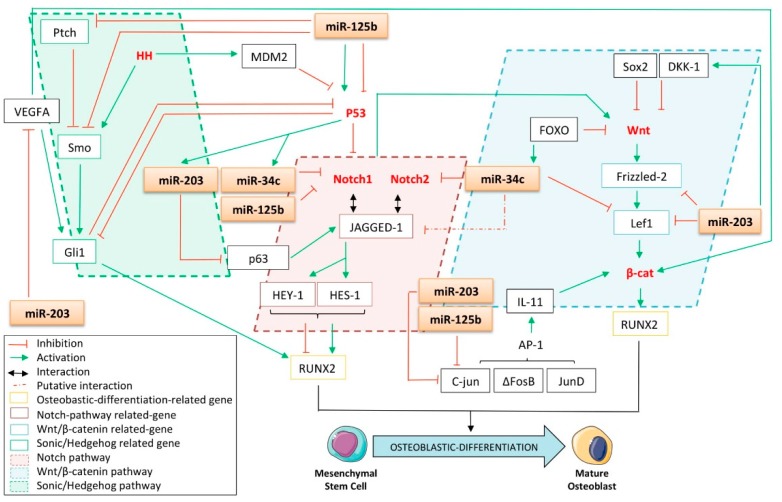
The p53-related miRNAs controls the osteoblastic differentiation through interplay with Notch, Wnt/β-catenin, and Sonic/Hedgehog (HH) pathways. AP-1: Activator protein-1; β-cat: β-catenin; DKK-1: Dickkopf Wnt signaling pathway inhibitor 1; FOXO: Forkhead transcription factor; HES-1: Hairy/enhancer f split; HEY-1: Hes-related protein; HH: Sonic/Hedgehog; IL: interleukin; Lef1: lymphoid enhancer-binding factor 1; MDM2: mouse double minute 2 homologue; RUNX2: runt-related transcription factor 2; Ptch: Patched receptor; Smo: Smoothened; VEGFA: Vascular endothelial growth factor A.

The Notch pathway, a transmembrane receptor, with its roles in development and cell fate determination is of particular interest in the context of this review. Its role throughout the course of both osteoblastic differentiation and tumorigenesis remains unclear and it seems that it has a dual effect on those processes, depending on the differentiation stage and cell context. For instance, it was reported that activation of the Notch pathway in osteocytes induces suppression of bone resorption in cancellous bones and an increased bone formation in cortical bones [121]. It was also demonstrated that such effects were mediated by the induction of the expression of osteoprotegerin (OPG) and by the activation of the Wnt/β-catenin pathway (Figure 4). These data were supported by Tezuka et al., who reported the early osteoblastic differentiation-promoting role of Notch1 in human primary bone marrow MSCs and its ability to stimulate calcified nodule formation in an MC3T3-E1 model [122]. In addition, *HES-1* (Hairy/enhancer of split) and *HEY-1* (Hes-related protein), two Notch-related genes, were reported to enhance the osteogenic differentiation of MSCs by cooperating with and inducing RUNX2′s expression [123]. Contradicting this data, genetic experiments have shown that removal of *Notch1* and *Notch2* from the early limb-mesenchyme of mice results in an increase in the trabecular bone mass of the animals, arguing in favor of an inhibitory role of such genes in osteoblastic differentiation [124]. In this work, investigators showed that this bone-phenotypic change was due to the Notch inhibitory effects on osteoblastic differentiation, at least as mediated by the HES and HEY proteins. In this case, the latter were found to directly interact with RUNX2 to consequently inhibit its transcriptional capabilities in both CHO and ST2 mouse bone stromal cells.

Furthermore, Notch signaling is often deregulated in cancers and, here, still has a dual role, as it supports either oncogenic or tumor suppressor functions, depending on the cancers [125]. Nonetheless, it seems that it favors osteosarcoma progression as an up-regulater of Notch itself. Up-regulation of JAGGED-1, HES-1, and HEY-2, three Notch target genes, and also the zinc finger transcription factor Osterix was reported in primary human osteosarcoma samples as compared to normal osteoblasts [126]. This data is enforced by the fact that Notch1, JAGGED-1, and Delta-like-1 were also found to be overexpressed in gliomas [127]. Regarding the effect of Notch signaling on osteoblastic differentiation and tumorigenesis, targeting this pathway could represent a relevant strategy toward cancer treatment, especially osteosarcoma. Additionally, Engin et al. showed an up-regulation of genes related to the Notch pathway in osteosarcoma samples developed in p53-heterozygous mice, suggesting that p53 is a Notch repressor in this context [126]. The multilevel crosstalk between both p53 and Notch pathways is well documented and occurs through reciprocal regulation of these two signaling factors [128]. Of note, the p53-inhibitory function on the Notch pathway described by Pastorcic et al. could be strengthened by the study of the p53-related miRNAs [129].

The putative targeting of Notch1 by miR-34c was first mentioned by Corney et al. and further confirmed by Bae’s work, who not only experimentally demonstrated the direct interaction between these two partners but also reported miR-34c’s inhibitory role throughout the course of the osteoblastic differentiation of bone marrow stromal cells [45,130]. Roy et al. also noticed a marked decrease in Notch1 protein expression consequent to the re-induction of miR-34c’s expression after the treating of colon cancer cells with difluorinated curcumin [36]. More recently, Xu et al. confirmed the targeting of Notch1 by miR-34c in an osteosarcoma model and reported that the inhibition of this miRNA promotes the chemoresistance of the cells [131]. In addition, it was reported that the expression of miR-34c is up-regulated during mouse spermatogenesis, although the expression of Notch1 was shown to be down-regulated [132]. Furthermore, Manca et al. outlined the direct involvement of miR-125b in Notch1 down-regulation through its Notch1-mRNA 3′UTR targeting (Figure 4) [133]. Investigators have also shown that the overexpression of miR-125b in human primary keratinocytes leads to a significant decrease in Notch1 at the protein level. In addition, Notch2 was identified by Bouhallier et al. as being a direct target of miR-34c in the HeLa cervical cancer cell line [134]. Wu et al. further found that only miR-34c-3p was able to reduce Notch2 protein expression in glioblastoma cells, whereas miR-34c-5p did not [33]. Furthermore, in silico approaches in mice genetic models predict the presence of a potential binding site for miR-34c in the 3′UTR of JAGGED-1, one of the Notch’s target genes, even if this has still not been experimentally validated [135]. Diao et al. recently reported that miR-203 indirectly impedes the Notch pathway through its targeting of the JAGGED-1-inducer p63 [56]. By introducing either miR-203 or a p63 siRNA into the RD rhabdomyosarcoma cell line, this group observed a reduction both in the JAGGED-1 transcript level and in HES-1 protein expression. Given the fact that both miR-125b and -34c are found to be underexpressed in osteosarcomas [24,72], the reintroduction of these miRNAs could provide a promising strategy against the spread of this osteoblast-derived tumor through impairment of the Notch pathway.

#### 4.1.2. The Wnt Pathway

The Wnt/β-catenin pathway was also reported as being of paramount importance in the biology of bones. Rawadi et al. were among the first to suggest the involvement of this pathway in the regulation of osteoblastic differentiation, by highlighting that a Wnt autocrine loop leads to the induction of ALP expression and mediates the BMP-2-induced mineralization of pre-osteoblasts [136]. These results were further supported by Mansukhani et al., who correlated the **Sox2**-inhibitory role on the Wnt pathway and the inhibition of osteoblastic differentiation [137]. In line with these considerations, it is interesting to note that Thatcher et al. found that miR-203 directly targets lymphoid enhancer-binding factor 1 (Lef1), one of the Wnt pathway-related players in a zebrafish model (Figure 4) [138]. Interestingly, Xu et al. also found that this gene is a target of miR-34c in an osteosarcoma model [131]. Moreover, recent evidence suggests the targeting of the Wnt/β-catenin-related receptor, Frizzled-2, by miR-203, even if no direct binding was attested [139]. In addition, it was found that miR-203 indirectly increases the expression of the Dickkopf Wnt signaling pathway inhibitor 1 (DKK-1), a secreted Wnt-related pathway inhibitor reported to impede osteogenesis in MSCs [140]. Interestingly, Lee et al. found that the expression of DKK-1 is increased in the sera of newly diagnosed osteosarcoma patients, suggesting the potential need to inhibit the Wnt/β-catenin pathway at the onset of osteosarcoma carcinogenesis [141]. Finally, it is known that the Forkhead transcription factor FOXO suppresses the Wnt pathway and bone formation [142], while FOXO was also reported to induce the expression of miR-34c, highlighting, here, another miR-34c-related way to repress osteoblastic differentiation.

The transcription factors ∆FosB and JunD are two partners of the activator protein-1 (AP-1) complex and are both implicated in osteoblastic differentiation through an indirect regulatory mechanism allowing for the proper expression of RUNX2 [143]. It was demonstrated that the ∆FosB/JunD complex mediates the transcription of interleukin-11 (IL-11), itself able to activate the Wnt/β-catenin pathway, rightly resulting in the induction of Runx2′s expression. In addition, miR-125b was identified as targeting c-Jun, another member of the AP-1 complex, in its coding region and was also found to be critical in reducing the proliferative potential and migration capabilities of melanoma cells [73]. Furthermore, c-Jun was identified by Sonkoly et al., Tripathi et al., and Luo et al. as a direct target of miR-203 [53,144,145].

#### 4.1.3. The Sonic/Hedgehog (HH) Pathway

The Sonic/Hedgehog (HH) pathway is involved in many aspects of development, including the control of osteoblastic differentiation. It is now well established that its deregulation also sustains the growth of many human tumors. It is important to note that p53 is known to inhibit HH signaling, at least through regulation of Gli1, one of the effectors of the HH pathway. It was shown that p53 was able to reduce Gli1′s expression level as well as its nuclear localization and activity [146]. In return, there is also a negative feedback loop exerted by HH on p53, as the same group demonstrated that Gli1 inhibits p53 (Figure 4) [146]. These data are strengthened by Abe et al., who demonstrated that HH induces the degradation of p53 by activating MDM2 [147]. It was found that miR-125b directly targets and functionally suppresses the seven-pass trans-membrane protein Smoothened (Smo), which rightly activates the Gli family of transcription factors [70]. Investigators have shown that the overexpression of miR-125b in medulloblastoma cells significantly reduces the mRNA levels of Gli1 and those of the Patched receptor (Ptch), both known as endogenous HH-related genes. In addition, the depletion of miR-125b increases Gli transcriptional activity, supporting the inhibitory role of this miRNA on the HH pathway (Figure 4). Vascular Endothelial Growth Factor A (VEGFA) is a growth factor implicated in the early stages of osteoblastic differentiation through its ability to stimulate the activity of the HH pathway and to increase the expression of β-catenin [148]. Interestingly, miR-203 was reported by Zhu et al. as targeting the VEGFA mRNA through its direct binding in a 3′UTR seed sequence conserved across species (Figure 4) [60]. This group also reported an up-regulation of VEGFA levels in cervical cancer patients, which is inversely correlated with the expression of miR-203. In addition, the overexpression of miR-203 in this model markedly suppresses cell proliferation, tumor growth, and angiogenesis.

### 4.2. The p53-Related miRNAs’ Interplay with Bone Morphogenetic Proteins (BMP) and Transforming Growth Factor-β (TGF-β) Pathways

#### 4.2.1. The Bone Morphogenetic Proteins (BMP) Pathway

**Figure 5:**
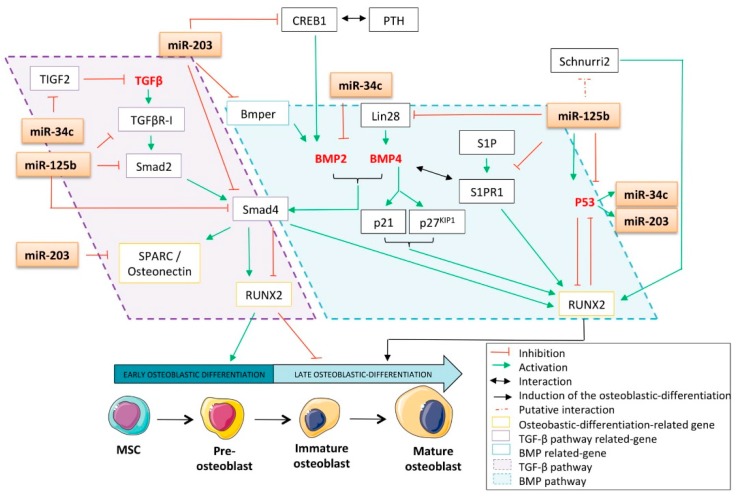
The p53-related miRNAs controls the osteoblastic differentiation through an interplay of both the bone morphogenetic protein (BMP) and the transforming growth factor β (TGF-β) pathways. **Bmper**: BMP endothelial cell precursor derived regulator; **BMP2**: bone morphogenetic protein 2; **BMP4**: bone morphogenetic protein 4; **CREB1**: c-AMP responsive element binding protein 1; **PTH**: parathormone; **RUNX2**: runt-related transcription factor 2; **SPARC**: secreted protein acidic and rich in cystein; **S1P**: Sphingosine 1 phosphate; **S1PR1**: Sphingosine 1 phosphate receptor 1; **TGF-β:** transforming growth factor β; **TGFβR-I:** transforming growth factor β geceptor I.

Bone Morphogenetic proteins (BMPs) are members of the Transforming Growth Factor-β (TGF-β) super-family of ligands and regulate various physiological processes including osteoblastic differentiation. BMPs are paracrine/autocrine local factors expressed by osteoblasts and they bind to two types of serine-threonine kinase receptors (BMPR-I and BMPR-II) to up-regulate RUNX2 expression during osteoblastic differentiation (Figure 5) [149]. The Smads proteins are the signaling effectors of both the BMP pathway and the canonical TGF-β pathway. They are classified into three subgroups related to their structures and functions [150]: the pathway-restricted Smads (R-Smads), the common-mediator Smad (C-Smad), and the inhibitory Smads (I-Smads). Smad4 is the lone C-Smad and is consequently implicated in the connection between the BMP and the TGF-β pathways. It is well-known that upon increase in the expression levels of the cyclin-inhibitors p21^CIP1^ and p27^KIP1^, BMP-4 induces a G_0_/G_1_ cell cycle arrest and enhances cell differentiation into osteoblast-like cells [151]. Thanks to their series of mice strains lacking one or several BMPs, Bandyopadhyay et al. showed that BMP2/BMP4-limb-deficient animals display critical defects in skeletal differentiation [152]. The distal femurs of mice that are one or three weeks of age do not show any bone marrow cavity, trabecular bone, or cortical bone as compared to their wild-type counterparts. This phenotype could be explained by the undifferentiated fibroblast-like osteoprogenitors found in the bone shaft of such mice, expressing normal amounts of Coll-I and RUNX2 but an insignificant amount of Osterix at three weeks. The work undertaken by these investigators reveals the particular role of BMP2 and BMP4 in the completion of osteogenesis.

Fotinos et al. recently reported that miR-34c directly targets the 3′UTR of BMP-2 in a lung carcinoma model, in which this miRNA was found to be down-regulated [153]. Cao et al. reported that the secreted glycoprotein BMP endothelial cell precursor derived regulator (Bmper) is a target of miR-203 (Figure 5) [154]. As Bmper is implicated in the sustained activation of the BMP pathway [155,156], the miR-203-targeting of this protein sustains its role against osteoblastic differentiation. In addition, targeting Bmper probably testifies to the miR-203 anti-tumor role, as this protein was also shown to be overexpressed in human colon, lung, and cervix adenocarcinomas and promotes cellular proliferative, migratory, and invasive behavior [155]. In addition, Saini et al. reported that human miR-203 binds to the 3′UTR of the C-Smad Smad4 in their prostate cancer model [54]. As Smad4 is the lone C-Smad, it is also implicated in mediating the TGF-β pathway, which was known to be involved in cancer progression and especially in the migratory features of malignant cells [157]. As a consequence, the targeting of Smad4 by miR-203 impairs both BMP and the TGF-β pathways and can modulate osteoblastic differentiation and/or tumorigenesis.

c-AMP responsive element-binding protein 1 (CREB1) is a transcription factor studied mainly in the neuronal context. Even if its exact functions in bone biology are not well understood, it is able to promote the differentiation of primary mouse osteoblasts. It was reported that the association of PTH with CREB1 promotes BMP-2 signaling, itself leading to the maturation of osteoblasts [158]. Interestingly, as assessed by RT-qPCR, Western blotting, and luciferase reporter assay, CREB1 was identified as a direct target of miR-203 in a multiple myeloma model (Figure 5) [55]. Of note, this specific targeting was further confirmed by Noguchi et al. in canine and human melanoma cells [159]. BMP-2 and TGF-β are able to modulate the expression of the secreted protein acidic and rich in cysteine matrix-associated protein (SPARC/osteonectin). It is interesting to note that this glycoprotein is a direct target of miR-203 [160] and that cell cultures from SPARC^-/-^ mice have a decreased number of osteoblasts associated with a decreased bone formation as compared to their wt counterparts [161]. It was recently described that sphingosine-1-phosphate receptors play a crucial role in osteoblastic differentiation, as S1P (sphingosine-1-phosphate) enhances both BMP-2-stimulated RUNX2 expression and the ALP activity of C2C12 cells [162]. Interestingly, it was reported that miR-125b directly inhibits sphingosine-1-phosphate receptor 1 (S1PR1) in the placenta [163]. Another possible BMP-related mechanism explaining the effect of miR-125b on osteoblastic differentiation could be the fact that the stem cell factor lin-28 is another validated target of this miRNA (Figure 5) [164]. Indeed, Ma et al. recently found that lin-28 was an inducer of the expression of BMP-4 [165]; therefore, miR-125b-mediated lin-28 inhibition could result in a lower BMP-4 level than that required for proper osteoblastic differentiation. Zhang et al. corroborated these results by comparing the miRNA expression profile of human adipose-derived stem cells (hADSC) pre- and post-osteogenic induction [166]. They found that miR-125b is down-regulated after osteogenic differentiation, whereas the zinc-finger transcription factor Schnurri-2, a putative target of this miRNA directly related to bone remodeling, was overexpressed [167].

#### 4.2.2. The Transforming Growth Factor-β (TGF-β) Pathway

Transforming Growth Factor-β (TGF-β) is a pleiotropic cytokine abundantly stored in bones. In mammals, it is found as three different isoforms: TGF-β1, TGF-β2, and TGF-β3. This cytokine has been reported to have a dual role during both osteoblastic differentiation and tumorigenesis; it has been widely reported that it promotes the early phases of osteoblastic differentiation and represses the terminal phases [168]. As TGF-β is reported to inhibit the expression of RUNX2 [169], it seems relevant that impairing the TGF-β pathway may have a critical influence on osteoblastic differentiation. This hypothesis was recently verified by Rana et al., who observed that impairing the TGF-β pathway through the use of an anti-TGF-β antibody improves doxorubicin-mediated inhibition of osteoblastic differentiation and increases the frequency of osteoblast colony-forming units [170]. In addition, the anti-osteoblastic-differentiation role of the TGF-β pathway was supported by Maeda et al. [171]. They demonstrated that the TGFβR-I inhibitor SB431542 not only promotes bone nodule formation from C2C12 mouse myoblasts but also increases both BMP-4-induced BSP expression and the production of ALP from human MSCs. Furthermore, TIGF2 is an inhibitor of the TGF-β pathway and was identified by Bouhallier et al. as being a direct target of miR-34c in a HeLa cervical cancer model [134]. MiR-34c-mediated TIGF2 inhibition could, thus, contribute to the activation of the TGF-β pathway and consequently result in the inhibition of osteoblastic differentiation. In addition, it was reported that the transfection of miR-125b into leukemia cells leads to a significant reduction in the expression of Smad2, Smad4, and TGFβR-I at the protein level [172]. These results were further sustained by the study of Zhou et al., who demonstrated the direct binding of miR-125 to the 3′-UTR of Smad2 [173].

### 4.3. The p53-Related miRNAs’ Interplay with Fibroblast Growth Factor (FGF), Epidermal Growth Factor (EGF), Janus Kinase/Signal Transducer and Activator of Transcription (JAK/STAT), C-jun N-Terminal Kinase/p38 (JNK/p38), the Mitogen-Activated Protein Kinase/ERK (MAPK/ERK) Pathway, and Other Kinase-Related Pathways

#### 4.3.1. The Fibroblast Growth Factor (FGF) Pathway

**Figure 6:**
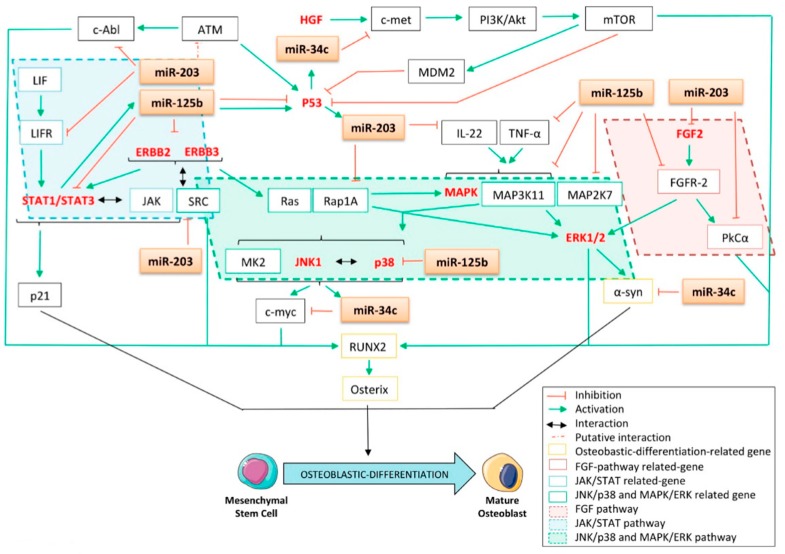
The p53-related miRNAs’ interplay with the fibroblast growth factor (FGF), the epidermal growth factor (EGF), the Janus kinase/signal transducer and activator of transcription (JAK/STAT), the C-jun N-terminal kinase/p38 (JNK/p38), the mitogen-activated protein kinase/ERK (MAPK/ERK) pathway and the other kinases-related pathways. ATM: ataraxia telangiectasia mutated; α-syn: α-synuclein; EGF: epidermal growth factor; FGF: fibroblast growth factor; FGF2: fibroblast growth factor 2; FGFR: fibroblast growth factor receptor; FGFR-2: fibroblast growth factor receptor 2; IL-22: interleukin-22; HGF: hepatocyte growth factor; JAK: Janus kinase; JNK: C-jun N-terminal kinase; JNK1: C-jun N-terminal kinase 1; LIF: leukemia inhibitory factor; LIFR: leukemia inhibitory factor receptor; MAPK: mitogen-activated protein kinase; MAP2K7: mitogen-activated protein kinase kinase 7; MAP3K11: mitogen-activated protein kinase kinase kinase 11; MDM2: mouse double minute 2 homologue; mTOR: mammalian target of rapamycin; PI3K: phosphoinositide 3 kinase; PKCα: protein kinase C alpha; STAT: signal transducer and activator of transcription; STAT1: signal transducer and activator of transcription 1; STAT3: signal transducer and activator of transcription 3; RUNX2: runt-related transcription factor 2; TNF-α: tumor necrosis factor α.

Fibroblast growth factor (FGF)/FGFR signaling is another crucial pathway involved in skeletal development [174]. Previously, a giant-cell tumor model demonstrated that FGFR-2IIIc signaling is a potent enhancer of the osteogenic differentiation program [175]. In this study, the authors showed that the knocking down of the expression of FGFR2-IIIc by siRNAs in mesenchymal stromal cells of a giant-cell tumor of the bone leads to a significant decrease in the expression of the three osteoblastic differentiation markers ALP, OPN, and RUNX2. Additionally, FGFR2-IIIc^-/-^ mice display delayed ossification during fetal development and dwarfism in the long bones and axial skeleton [176]. Moreover, a very recently published study reported that FGFR-1 is implicated in the proliferation of the MG63 osteosarcoma cell line, arguing for the inhibition of this pathway to potentially counteract the development of bone sarcomas [177]. It is, thus, interesting to correlate these data with the fact that miR-125b was shown to directly target FGFR-2 in a psoriasis model, suggesting another mechanism by which this miRNA could inhibit both osteoblastic differentiation and carcinogenesis (Figure 6) [75]. In addition, miR-203 was identified as being a direct inhibitor of FGF2 in renal cancer [178]. In accordance with these results, it was also highlighted that FGFR-2 promotes osteogenic differentiation through the serine/threonine kinase protein kinase C alpha (PKCα) and the ERK1/2 pathways in murine MSCs [179]. Interestingly, as miR-203 was validated as a bona fide direct regulator of PKCα in a lung cancer model (Figure 6) [180], the silencing of the expression of miR-34c was shown to activate PKC’s expression at the protein level, suggesting that this miRNA has an inhibitory role on osteoblastic differentiation [181].

#### 4.3.2. The Epidermal Growth Factor (EGF) Pathway

The implication of the epidermal growth factor receptor family member ERBB in periosteal osteoblastic differentiation was also reported [182]. In a bone-related cancer context, it was found that both the TC-71 and the SK-ES1 Ewing sarcoma cell lines overexpressed ERBB2 and that a blockade of this receptor led to increased sensitivity toward Taxol [183]. Furthermore, it was reported that miR-125b directly targets both ERBB2 and ERBB3 in SKBR3 breast cancer cells [184]. More recently, He et al. corroborated those results in ovarian cell lines stably overexpressing ERBB2 or ERBB3 lacking 3′UTR [74]. The results concerning ERBB2 were confirmed in umbilical vein endothelial cells [185], while the direct interaction between this mRNA and miR-125b was further confirmed in a small cell lung cancer model [186].

#### 4.3.3. The Janus Kinase/Signal Transducer and Activator of Transcription (JAK/STAT) Pathway

By generating mice with osteoblasts in which the expression of the signal transducer and activator of transcription 3 (STAT3) is knocked down, Itoh et al. reported that, in vivo, STAT3 signaling plays a critical role in bone formation because of the osteopenic phenotype that the animals display [187]. In line with these results, it was further demonstrated that STAT3 signaling promotes in vitro MSC osteoblastic differentiation in osteogenic media [188]. Interestingly, Bellido et al. showed that the induction of p21 is elicited by the binding of STAT3 at its promoter sequence (Figure 6) [189]. Furthermore, the G_0_/G_1_ cell-cycle arrest, required for committed osteoblast cells, is mediated by the induction of p21^CIP1^ in response to BMP4 [151]. In addition, siRNAs against p21 block OSM-induced ALP activity in the MG63 osteosarcoma cell line [189]. Together, this body of evidence suggests the role of p21 and STAT3 in osteoblastic differentiation and the proper function of osteoblast-derived cells. Thus, impairing STAT3 signaling through miRNA targeting could result in a decrease in osteoblastic differentiation.

The direct targeting of STAT3 by miR-125b was demonstrated by Liu et al. and could partially explain its inhibitory effect on osteoblastic differentiation (Figure 6) [72]. MiR-125b was found to be down-regulated in patients’ osteosarcoma samples as compared to the corresponding non-cancerous samples as well as in the MG63, SaOS-2, and U2OS osteosarcoma cell lines [72]. Another group confirmed that STAT3 is a miR-125b’s target by studying its implications during myelopoiesis [190]. In addition, as STAT3 is a well-known oncogene often overexpressed or activated in cancer [191], impairment in its expression could be a promising strategy toward cancer progression. By overexpressing miR-125b in the SaOS2 and MG-63 osteosarcoma cell lines, Liu et al. observed an in vitro decrease in cell growth and cell migration as well as delayed tumor growth in vivo [72]. Interestingly, this group has also shown that STAT3 is a transcriptional activator of the expression of miR-125b, highlighting a novel feedback loop regulation between STAT3 and miR-125b in osteosarcoma [72]. In addition, it is known that JAK1/STAT1/STAT3 signaling was activated through the binding of the leukemia inhibitory factor (LIF) to its receptor, LIFR. In this matter, it was demonstrated that the re-introduction of miR-203 into the RD and DH30 rhabdomyosarcoma cell lines leads to the subsequent inhibition of LIFR at the protein level (Figure 6) [56]. Furthermore, this group reports that, as a consequence of direct targeting of the LIFR transcript, miR-203 impairs proper JAK1/STAT1/STAT3 signaling activation. Together, these data could add a novel potential osteoblastic differentiation regulation mode through the p53/miR-203/STAT3 pathway.

#### 4.3.4. The C-jun N-Terminal Kinase/p38 (JNK/p38) and Mitogen-Activated Protein Kinase/ERK (MAPK/ERK) Pathways and Other Kinase-Related Pathways

Through their phosphorylating capabilities, kinase proteins are involved in the activation and/or repression of almost all the signaling cascades. This paragraph will focus on the clear evidence arguing for the interplay between p53-related miRNAs and such kinases, reinforcing the role of miRNAs as small inhibitors of osteoblastic differentiation. It is established that BMP-2-induced osteoblastic differentiation depends on both C-Jun N-terminal kinase (JNK) and p38 mitogen-activated protein kinase activation [192]. It was further clarified that such a differentiation process is sustained by the small G-protein Rap1A, through its ability to phosphorylate both ERK and p38, thus resulting in the activation of these pathways [193]. Interestingly, miR-125b was shown to directly target the 3′UTR of p38 [194] while miR-203 was reported to directly target the 3′UTR of Rap1A, adding here two other mechanisms by which these miRNAs reduce osteoblastic differentiation (Figure 6) [195]. In addition, it was recently demonstrated that the c-Myc proto-oncogene is a downstream target of JNK1 [196] as well as a proper inducer of osteogenesis, as its overexpression was reported to promote the BMP-2-induced osteoblastic differentiation of human MSCs [197]. Moreover, Cannell et al. reported that etoposide-induced DNA damage led to a significant down-regulation of c-Myc as well as the induction of the expression of miR-34c [198]. These authors correlated the results to miR-34c’s ability to directly target the c-Myc 3′UTR (Figure 6). Interestingly, they also found that the DNA-damage-mediated induction of miR-34c still occurred in SaOS2 osteosarcoma cells devoid of a functional p53, highlighting here an alternative p53-independent pathway involving p38/MAPK/MK2 leading to miR-34c transcription. Nonetheless, the interplay between the kinase pathways is complex, as mitogen-activated protein kinase kinase kinase 11 (MAP3K11) not only was reported to directly activate p38 but was also recently suggested to be miR-125b’s target in early B-cells [199]. Additionally, Xu et al. reported that the overexpression of miR-125b in the UT-SCC-7 cutaneous squamous carcinoma cell line consequently down-regulates the expression of mitogen-activated protein kinase kinase 7 (MAP2K7), leading to the inhibition of the G1/S transition and reducing both cellular proliferation and clonogenicity [71].

In line with these results, it was also shown that expression of the α-synuclein (α-syn) protein depends on the activation of the ERK1/2 pathway [200]. This protein further helps stimulate the differentiation of osteosarcoma cells, as its overexpression in the MG63 cell line leads to a significant increase in ALP activity as well as increased ALP and OCN expression [201]. Interestingly, miR-34c was reported to directly target the 3′UTR of the α-syn mRNA in human neuroblast cells (Figure 6) [202]. Similarly, the c-Abl tyrosine-kinase was also reported to be implicated in osteoblastic differentiation, as osteoblasts from stromal and calvarial explants isolated from Abl^-/-^ mice displayed a reduced expression of ALP and OCN associated with diminished mineral-deposition capabilities [203]. In line with this consideration, miR-203 was suggested to target c-Abl, as the protein expression level of the latter is inflected by an ectopic modulation of the expression of this miRNA [204]. In addition, it was shown that the kinase capabilities of the SRC protein implicated it in osteoblastic differentiation, as it helps stabilize Osterix, consequently increasing the transcriptional activity of this factor [205]. Interestingly, Wang et al. showed that miR-203 directly targets SRC in a lung cancer model (Figure 6) [206]. It was reported that the DNA-repair-associated Ser/Thr kinase Ataraxia telangiectasia mutated (ATM) is involved in osteoblastic differentiation, as it positively regulates the expression of Osterix [207]. Interestingly, a high expression of miR-203 was found to be significantly associated with a low expression of ATM in sporadic breast carcinomas [208].

Another interesting mechanism is worthy of attention here and concerns the reported synergistic effects of both the tumor necrosis factor alpha (TNF-α) and Interleukin-22 (IL-22) in inducing the phosphorylation of the MAPKs, p38, and ERK1/2 proteins in keratinocytes, thus resulting in the activation of those pathways [209]. Interestingly, in our bone-related concerns, Wang et al. reported the dual role of TNF-α throughout the course of the osteogenic differentiation of bone marrow MSCs [210]. Their study argues in favor of the promoting effect of this factor during the early phases of differentiation, while it seems to have an inhibitory role during the more advanced stages. The ability of miR-125b to directly bind to the TNF-α-3′UTR seems to correlate with its inhibitory effects on osteoblastic differentiation during the early stages of the process (Figure 6) [211]. In addition, even if ILs are a class of molecules implicated mainly in the immune response, they are also able to act on non-immune cells, thus sustaining several biological mechanisms such as differentiation. In particular, IL-22 was shown to promote the expression of some osteoblast-related markers such as RUNX2, MSX2, and OCN in cells from periodontal ligaments and to sustain the formation of mineralized nodules [212]. In this context, it is of particular interest that miR-203 directly inhibits the IL-22 mRNA (Figure 6) [213].

Finally, it was reported that enforcing the expression of the tyrosine kinase receptor c-met into primary cultures of human bone-derived cells converts them into osteosarcoma cells and leads to an increase in the expression of several osteoblastic differentiation markers such as RUNX2 and Osterix [214]. In addition, c-met was identified by Cai et al. as a direct target of miR-34c in a laryngeal carcinoma model (Figure 6) [30]. Investigators showed that increasing miR-34c’s expression in Hep-2 cells inhibits both proliferation and migrative capabilities as well as induces apoptosis. In addition, such c-met targeting was further confirmed in both gastric cancer [37] and uveal melanoma [32]. It was also reported, in this model, that miR-34c consequently impacts the p-Akt level in a hepatocyte growth factor (HGF)-dependent manner, further resulting in the inhibition of both the proliferative and migrative capabilities of the cells. In addition, Moumen et al. found that c-met enhances Phosphoinositide 3 kinase/Akt (PI3K/Akt) signaling, thus resulting in the activation of mammalian target of rapamycin (mTOR) [215]. As the latter exerts both a direct and an indirect inhibition of p53 through MDM2 activation, such a mechanism could explain the feedback-regulation loop between p53 and miR-34c.

### 4.4. The p53-Related miRNAs’ Interplay between Oxidative Stress, the Nuclear Factor-kappa b (NF-κb) Pathway, and Osteoblastic Differentiation

**Figure 7:**
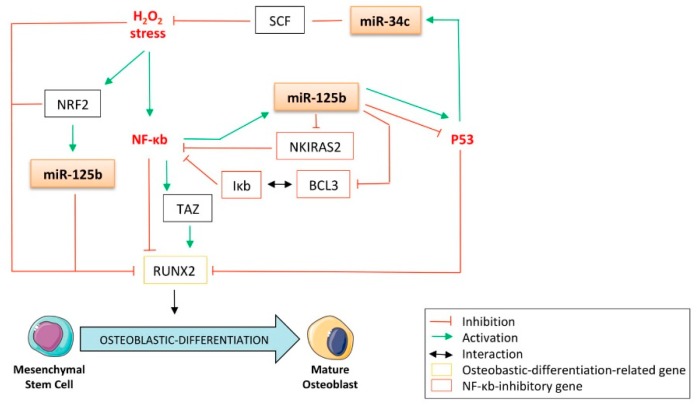
The p53-related miRNAs’ interplay between oxidative-stress, the nuclear factor-kappa b (NF-κb) pathway, and osteoblastic differentiation. BCL3: B-cell leukemia/lymphoma 3; Iκb: inhibitor of NF-κb; NKIRAS2: NF-κb Inhibitor interacting Ras-like 2; NF-κb: nuclear factor-kappa b; NRF2: nuclear factor (erythroid-derived 2)-like 2; RUNX2: runt-related transcription factor 2; SCF: stem cell factor; TAZ: tafazzin.

It was previously reported that H_2_O_2_ stress down-regulates the expression of the osteogenic markers ALP, BSP, and RUNX2, further resulting in the inhibition of osteoblastic differentiation of the MC3T3-E1 calvarial mouse osteoblast’s precursor cells [216]. These results are sustained by another study, in which osteoblasts treated with hydrogen peroxide, a reactive oxygen species (ROS)-generating agent, displayed a reduced expression of RUNX2 [217]. It was also demonstrated that oxidative stress inhibits osteoblastic differentiation via activation of the nuclear factor-kappa b (NF-κb) pathways [218]. Interestingly, the same study reported that NF-κb induces the transcription of miR-125b (Figure 7). These data corroborate the work of Manca et al., who found that hydrogen-peroxide-induced oxidative stress up-regulates miR-125b in HaCat keratinocytes [133]. In addition, it is interesting to note that the expression of miR-125b is sustained by the oxidative-stress-related transcription factor nuclear factor (erythroid-derived 2)-like 2 (NRF2) [219], which is rightly reported to impede osteoblastic differentiation through its inhibitory capabilities toward RUNX2-dependent transcriptional activity [220]. Furthermore, another control loop sustains the expression of miR-125b itself, thanks to its direct targeting capabilities towards NF-κb inhibitor interacting Ras-like 2 (NKIRAS2) [221], an NF-κb repressor [222]. It is also interesting to note that B-cell lymphoma/leukemia 3 (BCL3), which is a member of the NFκb-inhibitory complex Inhibitor of NF-κb (Iκb) [223], is another direct target of miR-125b (Figure 7) [224]. Thus, through those mechanisms, miR-125b still contributes to activating the NF-κb pathway, leading to the inhibition of osteoblastic differentiation. Intriguingly, contradictory data reports that NF-κb promotes the osteoblastic differentiation of human stromal cells derived from adipose tissue (hADSC) through induction of the transcriptional co-activator Tafazzin (TAZ) [225]. It was also demonstrated that miR-125b directly targets TAZ in hepatocellular carcinoma cells, suggesting another way by which this miRNA fine-tunes the maturation of the osteoblasts [226]. In line with this consideration, it was reported that the stem cell factor (SCF) protects the osteoblasts from oxidative stress [227] and that this factor is a direct target of miR-34c (Figure 7) [228].

## 5. The p53-Related miRNAs’ Interplay between the Epithelial-to-Mesenchymal Transition (EMT) and Osteoblastic Differentiation:

**Figure 8:**
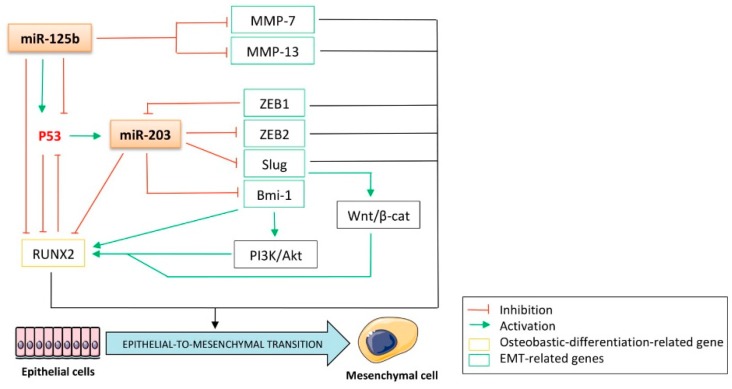
The p53-related miRNAs’ interplay between the epithelial-to-mesenchymal transition (EMT) and the osteoblastic-differentiation. **β-cat**: β-catenin; **EMT**: epithelial-to-mesenchymal transition; **MMP**: matrix metalloproteinase; **MMP-7**: matrix metalloproteinase 7; **MMP-13**: matrix metalloproteinase 13; **PI3K**: phosphoinositide 3 kinase; **RUNX2**: runt-related transcription factor 2.

The epithelial-to-mesenchymal transition (EMT) process is characterized by the loss of the epithelial features of the cancer cells, consequently triggering their mobility and spread throughout the body from the primary site in vivo. As the role of RUNX2 in osteoblastic differentiation and the osteogenic features of the bone-related cells is beyond any doubt, its implication in EMT was also reported [229]. The authors have shown that the siRNA-mediated silencing of this gene led to a significant down-regulation of the EMT markers Slug, Twist1, and matrix metalloproteinase 2 (MMP-2) in thyroid carcinoma cells. Bmi-1 is another important player in the chromatin-modulator polycomb complex involved in the promotion of the EMT process [230] and is often overexpressed in cancers. Its deregulation can partially explain some tumorigenic features of bone sarcomas, as it promotes both the in vitro anchorage-independent growth of Ewing sarcoma cells and their in vivo tumorigenicity [231]. It is further implicated in osteoblastic differentiation, as neonatal Bmi-1^-/-^ mice display skeletal growth retardation [232]. In addition, the expression of RUNX2 was reduced in the bone marrow MSCs from Bmi-1^-/-^ mice, which also exhibited decreased ALP activity. Wellner et al. were the first to report that the overexpression of miR-203 leads to Bmi-1-repression in a pancreatic carcinoma model due to its direct targeting of 3′UTR (Figure 8) [233]. These results were further confirmed by Saini et al. in a bone-metastatic prostate cancer model, in which the expression of miR-203 is attenuated [54]. In addition, Wu et al. recently showed that osteosarcoma cells overexpressing Bmi-1 are more prone to resist cisplatin through increased activation of the PI3K/Akt pathway and to display higher proliferative and migrative capabilities both in vitro and in vivo [234]. Furthermore, Yin et al. found that miR-203-mediated inhibition of Bmi-1 sensitizes breast cancer cells to 5-fluorouracil [235]. It was also found that re-expression of this miRNA in bone-metastatic prostate cancer cells induces the mesenchymal-to-epithelial transition (MET) characterized by increased E-cadherin expression and the down-regulation of the mesenchymal markers vimentin and fibronectin [54]. The functional consequences reported by the authors are the suppression of invasiveness and cell motility in vitro and a reduction in the metastatic potential in vivo, arguing in favor of the tumor-suppressive role of miR-203. Additionally, the E-box-binding transcriptional repressor ZEB2, a well-known E-cadherin-regulator and inducer of EMT [236], was found to be a direct target of miR-203 [54]. In turn, it was demonstrated that ZEB1 has a transcriptional inhibitory role toward miR-203 through its direct binding at its promoter region [233], suggesting a feedback loop between the ZEB family proteins and miR-203 (Figure 8). *Slug* is another interesting gene in the context of this review because of its implication in both the EMT process and the functioning of osteoblasts. Its expression was found to be correlated to the expressions of RUNX2, OPN, OCN, Coll-I, and the Wnt/β-catenin signaling in osteoblasts [237]. Interestingly, miR-203 was shown to be a bona fide inhibitor of this gene through its direct-targeting properties, as evidenced in a gastric carcinoma model (Figure 8) [238]. Given the metastatic spreading concerns of pathology, it is also important to note that Xu et al. reported the tumor-suppressive role of miR-125b through its ability to suppress the motility and invasive capabilities of cutaneous squamous carcinoma cells [71]. Their microarray and RT-qPCR analysis revealed that the overexpression of this miRNA in the UT-SCC-7 cutaneous squamous carcinoma cell line consequently down-regulates the expression of two matrix metalloproteinases (MMP):,MMP-7 and MMP-13, with the latter being further demonstrated as a direct target of miR-125b. This targeting was confirmed in a bladder cancer model [239]. In light of all this evidence, the p53-related miRNAs studied here display an interesting EMT-inhibitory role linked to their negative impact on osteoblastic differentiation.

## 6. The p53-Related miRNAs’ Control of the Cell Cycle and Apoptosis is Linked to Their Role as Osteoblastic-Differentiation Regulators

**Figure 9:**
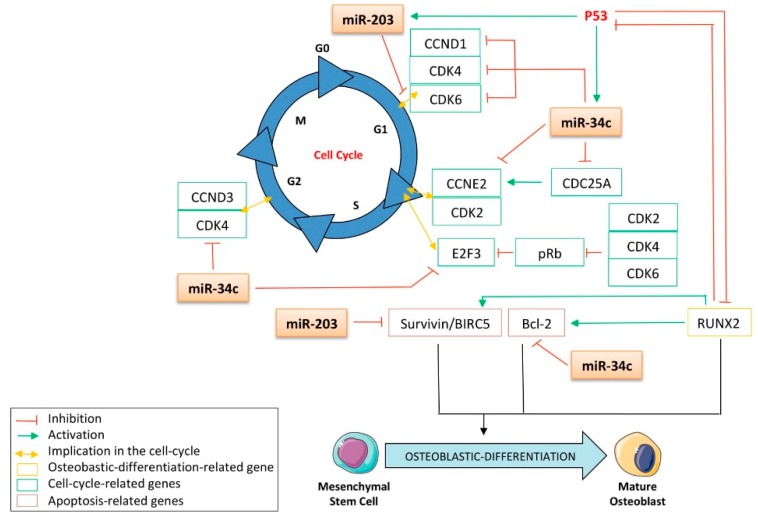
The p53-related miRNAs’ control of the cell cycle and apoptosis is linked to the role as osteoblastic-differentiation regulators. CCN: cyclin; CCND1: cyclin D1; CCND3: cyclin D3; CCNE2: cyclin E2; CDC25A: cell division cycle 25A; CDK: cyclin-dependent kinase; CDK2: cyclin-dependent kinase 2; CDK4: cyclin-dependent kinase 4; CDK6: cyclin-dependent kinase 6; Bcl-2: B-cell CLL/Lymphoma 2; RUNX2: runt-related transcription factor 2.

Regarding the amount of the aforementioned targets of miR-34c, -125b, and -203 involved in pathways directly or indirectly controlling osteoblastic differentiation, plenty of evidence has been highlighted of the implication of these miRNAs in the regulation of osteogenesis processes. Nonetheless, in controlling the cell proliferation rate and the apoptotic mechanisms, their tumor-suppressor role is of paramount interest in both a tumorigenicity context and a cellular-differentiation context. Interestingly, it was shown that the addition of calcium ions and vitamin D3 to the culture media of human alveolar bone explants promotes the expression of the small inhibitor of apoptosis (IAP) survivin/BIRC5 as well as several osteoblastic markers such as ALP and OCN, suggesting the implication of this gene in the maturation of osteoblasts [240]. By directly interacting with survivin, miR-203 was reported to inhibit the proliferation and induce the apoptosis of prostate cancer cells (Figure 9) [54]. The same targeting was further supported in a bladder cancer model [241]. Interestingly, ChIP assays in a prostate cancer model reveal that RUNX2 regulates survivin expression through its direct binding at consensus sequences in the survivin promoter [242]. Ji et al. reported the direct targeting of the anti-apoptotic factor B-cell CLL/Lymphoma 2 (Bcl-2) by miR-34c in the gastric cancer cell line Kato III [243]. Furthermore, ectopic transfections of this miRNA in the PC3 prostate cancer cell line lead to a significant reduction of the expression of Bcl-2 and E2F3 at the protein level, indicating that this miRNA also reduces the S-phase’s cell number [34]. It is also important to note that ChIP experiments and reporter assays reveal that RUNX2 was a direct inducer of Bcl-2 expression through its promoter-binding capabilities [244]. Furthermore, Nagase et al. demonstrated that the biological functions of the osteoblasts of Bcl-2^-/-^ mice were altered, thus highlighting the role of this gene in promoting the proper differentiation, activity, and survival of such a cell type [245].

Several publications have described the implication of the p53-related miRNAs in cyclin/cyclin-dependent kinase (CDK) regulation, thus resulting in a cell cycle blockade. The control of this process by these miRNAs is of particular interest, as it not only contributes to the death of the cancer cells but also leads to the inhibition of osteoblastic differentiation. For instance, ectopic transfections of miR-34c were reported to inhibit the growth of gastric cancer cells through suppression of the expression of cyclin-dependent kinase 4 (CDK4) and cyclin E2 (CCNE2) even if no direct interaction was demonstrated between the miRNA and these mRNAs (Figure 9) [37]. Wei et al. further confirmed that the same miRNA decreases the CDK4 and CDK6 levels in osteoblasts [97], while Dong et al. verified these results in uveal melanoma cells [32]. In addition, miR-34c was reported to directly target cyclin D1 (CCND1), thus helping to inhibit the proliferation of osteoblasts [97]. Similarly, the expression of CDK6 was consequently reduced to a miR-203 ectopic expression in both esophageal [246] and ovarian cancers [247]. In line with these data, it was also recently reported that the siRNA-mediated knockdown of cell division cycle 25A (CDC25A) inhibits the osteoblastic differentiation of hMSCs [248]. In addition, miR-34c was found to reduce the protein expression of CDC25A even if no direct interaction between this miRNA and the CDC25A mRNA was evidenced (Figure 9) [247].

## 7. Conclusions

Taken together, the results of all these studies validate or strengthen the notion that these miRNAs support p53′s counteracting activity against both osteoblastic differentiation and tumorigenesis. Such features are attributable to the huge variety of pathways in which their direct or indirect targets are involved, all contributing to the inhibition of the expression of RUNX2, an osteoblastic-differentiation-related gene regulating the very early steps of the differentiation process. Thus, as the p53-related miRNAs interfere with Notch, Wnt/β-cat, Sonic/Hedgehog (Figure 4), TGF-β, and BMP pathways (Figure 5), they have multiple ways to fine-tune the osteoblast-associated functions. Additionally, because they target pathways implicated in the control of proliferation, apoptosis, and cellular adaptation to the environment, such as the JAK/STAT, JNK/p38, and MAPK/ERK pathways (Figure 6), these miRNAs are obviously master regulators of tumorigenesis. Two of the p53-regulated miRs studied in this review have already been identified in bone or bone sarcomas (Table 1). Furthermore, as the different targets of these miRNAs is evidenced in both cancerous and non-cancerous models (Table S1), the universality of their action mode is highlighted here and might suggest that they could act together in our bone-related concerns. The various expression levels of these molecules during the course of the osteoblastic differentiation reveal that their target genes are necessarily subjected to a thin temporal control and remind us that the cellular fate is highly dependent of external signals. In the context of metabolic-bone pathologies, such as osteoporosis or osteopetrosis in which osteoblastic differentiation is deregulated, these miRNAs could provide interesting therapeutic options. In cancer-related concerns, it seems that these miRNAs control the pathways implicated in oxidative stress to which the cancer cells are subjected during the tumor-growth process (Figure 7), as well as the EMT, which allows them to further disseminate into the body (Figure 8). Finally, these miRNAs are also implicated in cell-cycle regulation in which p53 plays a major role (Figure 9). However, as p53 is often mutated in osteosarcoma and in Ewing sarcoma, it seems obvious that alternative pathways lead to the expression of these miRNAs and promote their anti-tumor role in the absence of this factor. Collectively, these data construct a novel glimpse at the role of these miRNAs as promising targets for improving bone-associated disease treatments, especially in osteosarcomas, which combine defects in both the osteoblastic-differentiation program and in the control of normal cellular behavior.

## Figures and Tables

**Table 1:** p53-related miRNAs and the nature of the interaction with their target genes in bone development and sarcomas.

Characterisation of the Interaction					Reference		
miRNA	p53’s Link	Target Gene	Nature of the Targeting	Model	Authors	Date of Publication	Journal
**miR-125b**	conflicting data: activator of p53’s expression & repressor of p53’s expression	**CBFβ**	direct 3’UTR	Mesenchymal stem cells	Huang et al.,	2014	Biochimie
		**p53**	direct 3’UTR	Ewing Sarcoma	Ida et al.,	2013	Cancer Cell Int.
		**STAT3**	direct 3’UTR	Osteosarcoma	Liu et al.,	2011	Biochem Biophys Res Commun
**miR-34c**	directly induced by p53	**CCND1**	direct 3’UTR	Mouse osteoblasts	Wei et al.,	2012	J Cell Biol.
		**CDK4**	no direct 3’UTR	Mouse osteoblasts	Wei et al.,	2012	J Cell Biol.
		**CDK6**	no direct 3’UTR	Mouse osteoblasts	Wei et al.,	2012	J Cell Biol.
		**Lef1**	direct 3’UTR	Osteosarcoma	Xu et al.,	2014	Med Oncol.
		**Notch1**	direct 3’UTR	Osteosarcoma	Xu et al.,	2014	Med Oncol.
		**RUNX2**	direct 3’UTR	Mouse osteoblasts	Zhang et al.,	2012	J Biol Chem.
			direct 3’UTR	Osteosarcoma	Van der Deen et al.,	2013	J Biol Chem.
		**SATB2**	direct 3’UTR	Mouse osteoblasts	Wei et al.,	2012	J Cell Biol.
		**TRPS1**	direct 3’UTR	Mouse osteoblasts	Zhang et al.,	2012	J Biol Chem.

## Supplementary Materials

The following are available online at https://www.mdpi.com/2073-4409/9/4/810/s1, Table S1: The p53-related miRNAs and the nature of the interaction with their target genes.
