# Supplementary material for: Implication of the p53-Related miR-34c, -125b, and -203 in the Osteoblastic Differentiation and the Malignant Transformation of Bone Sarcomas
Source: Cells. 2020 Mar 27;9(4):810. doi: 10.3390/cells9040810 (PMC7226610; doi:10.3390/cells9040810)
Supplement: Supplementary file 1 [file cells-09-00810-s001.pdf]

characterisation of the interaction					reference		
miRNA	p53's link	target gene	nature of the targeting	model	authors	date of publication	Journal
miR-125b	conflicting data: activator of p53's expression & repressor of p53's expression	Bcl-3	direct 3'UTR	Ovarian cancer	Guan et al.,	2011	Int J Cancer
		CBFβ	direct 3'UTR	Mesenchymal stem cells	Huang et al.,	2014	Biochimie
		C-Jun	direct 3'UTR	Melanoma	Kappelmann et al.,	2013	Oncogene
		ERbB2	direct 3'UTR	Breast cancer	Scott et al.,	2007	J Biol Chem.
			direct 3'UTR	Ovarian cancer	He et al.,	2013	PLoS One
			no direct 3'UTR	Human Umbilical Vein Endothelial Cells	Zhou et al.,	2015	Burns
			direct 3'UTR	Small cell lung cancer	Yagishita et al.,	2015	Mol Cancer Ther.
		ERbB3	direct 3'UTR	Breast cancer	Scott et al.,	2007	J Biol Chem.
			direct 3'UTR	Ovarian cancer	He et al.,	2013	PLoS One
		FGFRII	direct 3'UTR	Psoriasis model	Xu et al.,	2011	J Invest Dermatol.
		Gli1	no direct 3'UTR	Medulloblastoma	Ferretti et al.,	2006	EMBO J.
		Lin -28	direct 3'UTR	Embryonnal carcinoma cells	Wu et al.,	2005	Mol Cell Biol.
		MAPK27	no direct 3'UTR	Cutaneous squamous cell carcinoma	Xu et al.,	2012	J Biol Chem
		MAP3K11	no direct 3'UTR	Early B-cells	Knackmuss et al.,	2015	Cell Death Differ.
		MMP7	no direct 3'UTR	Cutaneous squamous cell carcinoma	Xu et al.,	2011	J Biol Chem
		MMP13	direct 3'UTR	Cutaneous squamous cell carcinoma	Xu et al.,	2012	J Biol Chem
			direct 3'UTR	Bladder cancer	Wu et al.,	2013	Oncol Lett.
		NKIRAS2	direct 3'UTR	Glioblastoma	Haemmig et al.,	2014	Cell Death Dis.
		Notch1	direct 3'UTR	Human primary keratinocytes	Manca et al.,	2011	Exp Dermatol.
		Patched	no direct 3'UTR	Medulloblastoma	Ferretti et al.,	2007	EMBO J.
		$p14^{ARF}$	direct 3'UTR	Prostate cancer	Amir et al.,	2013	PLoS One
		p38	direct 3'UTR	Human keratinocytes, human embryonic kidney cells	Tan et al.,	2012	J Biol Chem
		p53	direct 3'UTR	Human lung fibroblasts, zebrafish brain	Le et al.,	2009	Genes Dev.
			direct 3'UTR	Ewing Sarcoma	Ida et al.,	2013	Cancer Cell Int.
		RUNX2	no direct 3'UTR	Rat thoracic aorta	Chen et al.,	2013	PLoS One
		Schnurri-2	no direct 3'UTR	Human adipose-derived stem cells	Zhang et al.,	2012	J Cell Biochem.
		Smad2	no direct 3'UTR	Leukemia	Emmrich et al.,	2014	Genes Dev.
			direct 3'UTR	Hepatocellular carcinoma	Zhou et al.,	2015	Hepathology
		Smad4	no direct 3'UTR	Leukemia	Emmrich et al.,	2014	Genes Dev.
		Smoothened	direct 3'UTR	Cerebellar neuronal progenitors cells, medulloblastoma	Ferretti et al.,	2008	EMBO J.
		STAT3	direct 3'UTR	Murine bone marrow cells	Surdziel et al.,	2011	Blood
			direct 3'UTR	Osteosarcoma	Liu et al.,	2011	Biochem Biophys Res Commun
		S1P	direct 3'UTR	Placenta	Li et al.,	2014	Biochem Biophys Res Commun
		TAZ	direct 3'UTR	Hepatocellular carcinoma	Li et al.,	2015	Oncol Lett.
		TGFβR-I	no direct 3'UTR	Leukemia	Emmrich et al.,	2014	Genes Dev.
		TNF-α	direct 3'UTR	LPS-stimulated macrophages	Kim et al.,	2015	Immunol Invest.
miR-34c	directly induced by p53	α-syn	direct 3'UTR	Human neuroblast cells	Kabaria et al.,	2015	FEBS Lett.
		Bcl-2	direct 3'UTR	Gastric cancer	Ji et al.,	2008	BMC Cancer
			no direct 3'UTR	Prostate cancer	Hagman et al.,	2010	Int J Cancer
		BMP-2	direct 3'UTR	Lung cancer	Fotinos et al.,	2014	Anticancer Res.
		CCND1	direct 3'UTR	Mouse osteoblasts	Wei et al.,	2012	J Cell Biol.
		CCNE2	no direct 3'UTR	Gastric cancer	Suzuki et al.,	2011	Carcinogenesis
		CDC25A	no direct 3'UTR	Ovarian cancer	Yuan et al.,	2015	Int J Clin Exp Med.
		CDK4	no direct 3'UTR	Gastric cancer	Suzuki et al.,	2010	Carcinogenesis
			no direct 3'UTR	Mouse osteoblasts	Wei et al.,	2012	J Cell Biol.
			no direct 3'UTR	Uveal melanoma	Dong et al.,	2012	Mol Vis.
		CDK6	no direct 3'UTR	Mouse osteoblasts	Wei et al.,	2012	J Cell Biol.
			no direct 3'UTR	Uveal melanoma	Dong et al.,	2012	Mol Vis.
			no direct 3'UTR	Ovarian cancer	Yuan et al.,	2015	Int J Clin Exp Med.
		c-met	no direct 3'UTR	Gastric cancer	Suzuki et al.,	2010	Carcinogenesis
			direct 3'UTR	Laryngeal carcinoma model	Cai et al.,	2010	Int J Mol Med.
			direct 3'UTR	Uveal melanoma	Dong et al.,	2012	Mol Vis.
		c-myc	direct 3'UTR	Cervical cancer	Cannell et al.,	2010	Proc Natl Acad Sci USA
		E2F3	no direct 3'UTR	Prostate cancer	Hagman et al.,	2011	Int J Cancer
		JAGGED-1	no direct 3'UTR	Mouse	Hoesel et al.,	2010	Gene Expr Patterns.
		Lef1	direct 3'UTR	Osteosarcoma	Xu et al.,	2014	Med Oncol.
		Notch1	no direct 3'UTR	Primary mouse ovarian surface epithelium cells	Corney et al.,	2007	Cancer Res.
			no direct 3'UTR	Colon cancer	Roy et al.,	2012	J Hematol Oncol.
			direct 3'UTR	Bone marrow stromal cells	Bae et al.,	2012	Hum Mol Genet.
			no direct 3'UTR	Mouse teste cells	Sree et al.,	2014	Biol Reprod.
			direct 3'UTR	Osteosarcoma	Xu et al.,	2014	Med Oncol.
		Notch2	direct 3'UTR	Cervical cancer	Bouhallier et al.,	2010	RNA
			direct 3'UTR	Glioblastoma	Wu et al.,	2013	Oncol Lett.
		PKC	no direct 3'UTR	Hippocampal cells from mice	Cao et al.,	2014	Cell Biol Int.
		RUNX2	direct 3'UTR	Mouse osteoblasts	Zhang et al.,	2012	J Biol Chem.
			direct 3'UTR	Osteosarcoma	Van der Deen et al.,	2013	J Biol Chem.
		SATB2	direct 3'UTR	Mouse osteoblasts	Wei et al.,	2012	J Cell Biol.
		SCF	direct 3'UTR	Vascular smooth muscle cells	Choe et al.,	2015	Cell Signal.
		TIGF2	direct 3'UTR	Cervical cancer	Bouhallier et al.,	2010	RNA
		TRPS1	direct 3'UTR	Mouse osteoblasts	Zhang et al.,	2012	J Biol Chem.
miR-203	indirectly induced by p53	ATM	no direct 3'UTR	Sporadic breast carcinomas	Rondeau et al.,	2015	Br J Cancer
		Bmi-1	direct 3'UTR	Pancreatic carcinoma	Wellner et al.,	2009	Nat Cell Biol.
			no direct 3'UTR	Prostate cancer	Saini et al.,	2011	Clin Cancer Res.
			direct 3'UTR	Breast cancer	Yin et al.,	2013	PLoS One
		Bmper	direct 3'UTR	Mouse epithelial stem cells	Cao et al.,	2013	Development
		c-Abl	no direct 3'UTR	Smooth muscle cells	Liao et al.,	2015	Physiol Rep.
		CDK6	no direct 3'UTR	Oesophageal cancer	Zhang et al.,	2014	J Cell Physiol.
			no direct 3'UTR	Ovarian cancer	Yuan et al.,	2015	Int J Clin Exp Med.
		c-Jun	direct 3'UTR	Basal cell carcinoma	Sonkoly et al.,	2012	Oncogenesis
			direct 3'UTR	Chicken fibroblasts	Luo et al.,	2014	Cell Death Dis.
			direct 3'UTR	Cortical neuron cells	Tripathi et al.,	2014	J Biomed Sci.
		CREB1	direct 3'UTR	Multiple myeloma	Wong et al.,	2011	Br J Haematol.
			direct 3'UTR	Canine and human melanoma	Noguchi et al.,	2014	Vet Comp Oncol.
		Dlx5	direct 3'UTR	Prostate cancer	Saini et al.,	2011	Clin Cancer Res.
		FGF2	direct 3'UTR	Renal cancer	Xu et al.,	2015	Diagn. Pathol.
		Frizzled-2	no direct 3'UTR	Lung carcinoma	Mine et al.,	2015	PLoS One
		HES1	no direct 3'UTR	Rhabdomyosarcoma	Diao et al.,	2014	J Biol Chem.
		IL-22	direct 3'UTR	Oral biopsies from Oral Lichen Planus	Shen et al.,	2015	J Oral Pathol Med.
		JAGGED-1	no direct 3'UTR	Rhabdomyosarcoma	Diao et al.,	2014	J Biol Chem.
		Lef1	direct 3'UTR	Zebrafish	Thatcher et al.,	2008	Proc Natl Acad Sci USA
		LIFR	no direct 3'UTR	Rhabdomyosarcoma	Diao et al.,	2014	J Biol Chem.
		PKCα	direct 3'UTR	Lung cancer	Wang et al.,	2013	PLoS One
		p63	direct 3'UTR	Human, mouse, zebrafish	Yi et al.,	2008	Nature
		Rap1A	direct 3'UTR	Prostate cancer	Xiang et al.,	2015	J Exp Clin Cancer Res.
		RUNX2	direct 3'UTR	Prostate cancer	Saini et al.,	2011	Clin Cancer Res.
		Slug	direct 3'UTR	Gastric carcinoma	Shi et al.,	2015	Tumour Biol.
		Smad4	direct 3'UTR	Prostate cancer	Saini et al.,	2011	Clin Cancer Res.
		SPARC	direct 3'UTR	Head and neck squamous cell carcinoma	Benaich et al.,	2014	Cell rep.
		SRC	direct 3'UTR	Lung cancer	Wang et al.,	2014	PLoS One
		Survivin	direct 3'UTR	Prostate cancer	Saini et al.,	2011	Clin Cancer Res.
			direct 3'UTR	Bladder cancer	Zhang et al.,	2015	PLoS One
		VEGFA	direct 3'UTR	Cervical cancer	Zhu et al.,	2013	Cell Physiol. Biochem.
		ZEB2	direct 3'UTR	Prostate cancer	Saini et al.,	2011	Clin Cancer Res.

Supplementary Table S1. The p53-related miRNAs and the nature of the interaction with their target genes. The p53-related miRNAs and their target genes are summarized and the target genes are classified in alphabetical order. The nature of the interaction is defined as a direct/indirect interaction between the miRNA and the target mRNA. A direct interaction is shown as validated if the authors performed luciferase assay through a 3′UTR-reporter system. The authors, the date of publication, and the journals are also listed among all the references of this review.
